# Fragment-Based Quantum Mechanical Calculation of Excited-State Properties of Fluorescent RNAs

**DOI:** 10.3389/fchem.2021.801062

**Published:** 2021-12-22

**Authors:** Chenfei Shen, Xianwei Wang, Xiao He

**Affiliations:** ^1^ Shanghai Engineering Research Center of Molecular Therapeutics and New Drug Development, School of Chemistry and Molecular Engineering, East China Normal University, Shanghai, China; ^2^ College of Science, Zhejiang University of Technology, Hangzhou, China; ^3^ New York University-East China Normal University Center for Computational Chemistry at New York University Shanghai, Shanghai, China

**Keywords:** fluorescent RNA, fragment-based quantum mechanical method, excited-state properties, molecular dynamics simulation, molecular modelling

## Abstract

Fluorescent RNA aptamers have been successfully applied to track and tag RNA in a biological system. However, it is still challenging to predict the excited-state properties of the RNA aptamer–fluorophore complex with the traditional electronic structure methods due to expensive computational costs. In this study, an accurate and efficient fragmentation quantum mechanical (QM) approach of the electrostatically embedded generalized molecular fractionation with conjugate caps (EE-GMFCC) scheme was applied for calculations of excited-state properties of the RNA aptamer–fluorophore complex. In this method, the excited-state properties were first calculated with one-body fragment quantum mechanics/molecular mechanics (QM/MM) calculation (the excited-state properties of the fluorophore) and then corrected with a series of two-body fragment QM calculations for accounting for the QM effects from the RNA on the excited-state properties of the fluorophore. The performance of the EE-GMFCC on prediction of the absolute excitation energies, the corresponding transition electric dipole moment (TEDM), and atomic forces at both the TD-HF and TD-DFT levels was tested using the Mango-II RNA aptamer system as a model system. The results demonstrate that the calculated excited-state properties by EE-GMFCC are in excellent agreement with the traditional full-system time-dependent *ab initio* calculations. Moreover, the EE-GMFCC method is capable of providing an accurate prediction of the relative conformational excited-state energies for different configurations of the Mango-II RNA aptamer system extracted from the molecular dynamics (MD) simulations. The fragmentation method further provides a straightforward approach to decompose the excitation energy contribution per ribonucleotide around the fluorophore and then reveals the influence of the local chemical environment on the fluorophore. The applications of EE-GMFCC in calculations of excitation energies for other RNA aptamer–fluorophore complexes demonstrate that the EE-GMFCC method is a general approach for accurate and efficient calculations of excited-state properties of fluorescent RNAs.

## Introduction

RNA directly regulates a large number of cellular processes, and effective methods are desirable to fluorescently label and track RNA in living cells (Autour et al., 2018). Because RNA lacks inherent fluorescence, it is difficult to track RNA molecules in real time. (Dolgosheina et al., 2014). Unrau and coworkers demonstrated that the high binding affinity of RNA Mango to its fluorophore provided a useful tool for single-molecule RNA visualization and for fluorescently monitoring RNA complexes while simultaneously using the fluorophore as a purification tag (Dolgosheina et al., 2014). Fluorescent aptamers have been successfully applied to track and tag RNA in a biological system, but the excited-state properties of the RNA aptamer–fluorophore complexes can hardly be predicted by the traditional quantum mechanical (QM) methods due to their large molecular size (Raghavachari and Saha, 2015; Li et al., 2016; Liu et al., 2017; Nakai and Yoshikawa, 2017).

**FIGURE 1:**
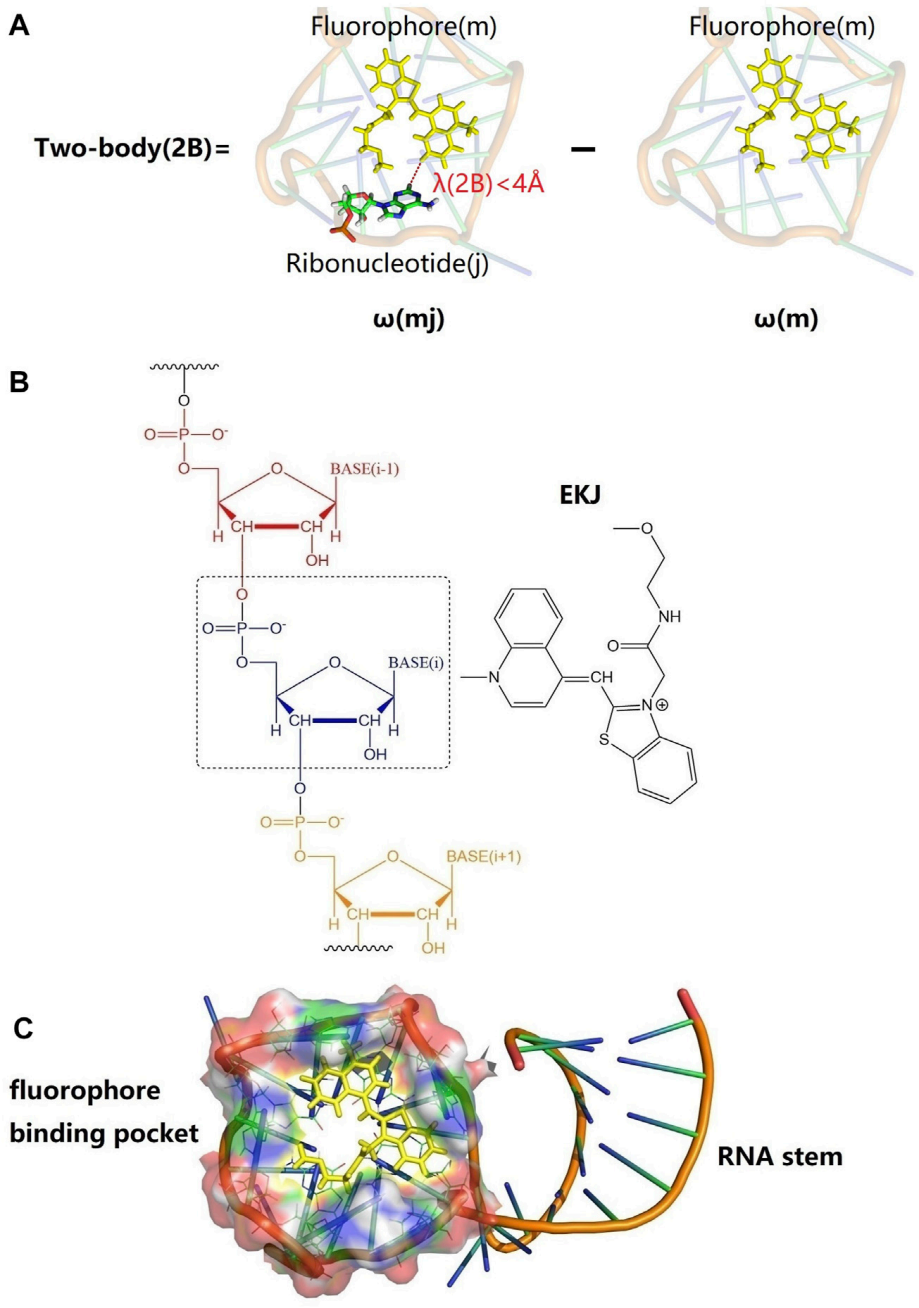
Graphical representations of fluorophore (EKJ) and Mango II RNA aptamer (PDB id: 6C63) and illustration of the two-body (2B) treatment of the excitation calculation in the EE-GMFCC fragmentation method for RNA systems. **(A)** An example of 2B calculation; the yellow stick represents the locally excited region of the fluorophore (*m*). A22 is a ribonucleotide in the RNA, whose spatial distance from the fluorophore is within a predefined threshold of λ(2B) (here 4 Å was employed). **(B)** The EE-GMFCC scheme where the bond is cut for the *i*th ribonucleotide (in black dotted box), and the *i*th fragment was utilized in two-body QM calculation with the EKJ. **(C)** Mango II-EKJ complex. Mango II RNA consists of two parts: a stem and fluorophore-binding pocket.

Mango II is an RNA aptamer that can accurately image the subcellular localization of three small noncoding RNAs in fixed and live mammalian cells (Autour et al., 2018; Trachman et al., 2018). These aptamers normally contain a closing RNA stem as shown in Figure 1C, which isolates a small fluorophore-binding core from the external sequence, making them easy to insert into arbitrary biological RNA (Trachman et al., 2019). Usually, the fluorophore would bind to RNA with non-covalent interactions.

Many QM methods were proposed for excited-state calculations, such as the approximate coupled cluster singles and double (CCSD) model (CC2) (Christiansen et al., 1995), symmetry-adapted cluster configuration interaction (SAC-CI) (Nakatsuji, 1979), complete active space second-order perturbation theory (CASPT2) (Finley et al., 1998), equation-of-motion CCSD (EOM-CCSD) (Stanton and Bartlett, 1993), time-dependent Hartree–Fock (TD-HF) (McLachlan and Ball, 1964), configuration interaction singles (CIS) (Foresman et al., 1992), and time-dependent density functional theory (TD-DFT) (Gross and Kohn, 1990; Adamo and Jacquemin, 2013; Kocherzhenko et al., 2017). Nevertheless, the applications of such methods to large molecules, such as proteins and RNAs, suffer from the limitations of expensive computational cost.

In order to reduce the computational cost of the excited-state calculations, a series of approaches were proposed for the systems with a localized electronic excitation (Jin et al., 2020). The simplest treatment is on the basis of the QM/MM method, which only apply the high-level QM method to the fluorophore, while the rest of the system was modeled with an empirical molecular mechanical (MM) method (Dahlke and Truhlar, 2007; Khait and Hoffmann, 2010; Kluner et al., 2011; Isborn et al., 2012; Daday et al., 2013; Milanese et al., 2017). More sophisticated approaches are using fragmentation QM techniques, which seek to reproduce full-system QM calculations by taking a proper combination of calculations of a series of individual fragments (Collins and Bettens, 2015; Scholz and Neugebauer, 2021).

Some fragmentation QM approaches have been proposed for calculations of the excited-state properties of large systems, including the generalized energy-based fragmentation (GEBF) approach developed by Li and coworkers (Li et al., 2016), the divide-and-conquer (D and C) method of Nakai and coworkers (Yoshikawa et al., 2013), the extension of the binary-interaction method (Hirata et al., 2005) of Hirata et al., and the fragment molecular orbital (FMO) method of Kitaura and coworkers (Chiba et al., 2007; Nakata et al., 2014). Recently, the electrostatically embedded generalized molecular fractionation with conjugate caps (EE-GMFCC) method was developed to calculate the excited-state properties of molecular crystals (Liu et al., 2019; Zhang et al., 2020) and fluorescent proteins (Jin et al., 2020) by our group. In this work, the EE-GMFCC method was further extended to predict the excited-state properties of fluorescent RNA–aptamer systems.

This paper is organized as follows. First, the convergence of the EE-GMFCC excitation energy as a function of the distance threshold for two-body QM interactions was tested for fluorescent RNA–aptamer systems. Second, the accuracy of the EE-GMFCC method on prediction of the excitation energy was investigated at both TD-HF and TD-DFT levels by comparison with the traditional full-system calculations. Third, the accuracy of calculated transition electric dipole moments (TEDM) and atomic forces by the EE-GMFCC method was demonstrated. Furthermore, a 100-ns molecular dynamics (MD) simulation of Mango II RNA in explicit water solvent was performed, and the relative excited-state energies of 10 different configurations extracted from the MD simulation were calculated by EE-GMFCC and compared with the results obtained from corresponding full-system QM calculations. Finally, the performance of the EE-GMFCC approach was extensively assessed on some other fluorescent RNA–aptamer systems by direct comparison with the full-system QM calculations.

## Computational Approaches

Our previous work showed that the QM effects from the protein environment played a significant role in the calculations of the excited-state properties of green fluorescent protein (GFP) (Creemers et al., 1999; Creemers et al., 2000; Jin et al., 2020). Therefore, the local chemical environment was expected to be treated by the electronic structure method for accurately capturing the QM effects. Herein, the fragment-based QM method EE-GMFCC was proposed for incorporating the environmental effects of RNA on calculation of the excited-state properties of chromophore in the RNA aptamer–fluorophore complex. The EE-GMFCC method is an extension of the GMFCC/MM approach (He and Zhang, 2006). In the GMFCC/MM method, a system (proteins or RNA) would be divided into a series of unit-based fragments, and the properties, such as the total energy of the system, were obtained by taking a proper combination of the QM properties of subsystems. Generally, two-body interaction energy calculations were performed to capture the QM effects between the non-neighboring units that are spatially in close contact within a predefined distance threshold (Jin et al., 2017; Liu et al., 2017; Liu and He, 2017; Wang et al., 2018; Liu et al., 2019; Zhang et al., 2020). For further accounting for higher-order many-body electrostatic effects, an electrostatically embedding scheme was employed in the EE-GMFCC method by using embedding charges representing the remaining fragments in each fragment QM calculation (Wang et al., 2013; Jin et al., 2017). For clarity, before describing the application of the EE-GMFCC method in the calculation of the excited state of the RNA aptamer–fluorophore complex, we would give a brief description of the EE-GMFCC method for calculating the ground-state energies of RNAs.

### The EE-GMFCC Method for Calculation of Ground-State Energy of RNA

The EE-GMFCC method was initially developed for calculations of the ground-state total energies of proteins (Wang et al., 2013; He et al., 2014; Jin et al., 2017; Liu and He, 2020). In the framework of the EE-GMFCC method, a protein with *N* residues is divided into *N*-2 fragments with each residue capped by its neighboring residues (conjugate caps) (Wang et al., 2013; Jin et al., 2017; Wang et al., 2018), and then the total energy of the given protein is obtained *via* taking a proper combination of the QM-calculated energies of the neighboring residues. For accurately capturing the QM effect between non-neighboring residues in close contact (within a predefined distance threshold λ), the corresponding two-body interactions are also treated at the QM level. Generally, higher-order interactions within the EE-GMFCC scheme are small and can be neglected due to the electrostatic embedding treatment.

Similar to the treatment of the protein, EE-GMFCC can be utilized in the calculation of the ground-state energy of a given RNA system. The ground-state energy of a RNA system is calculated using the EE-GMFCC method as follows (Jin et al., 2017),

$$\begin{array}{l} E_{\text{EE}-\text{GMFCC}}^{\text{Ground state}}\;\;=\;\;E_{\text{MFCC}}+E_{\text{ribonucleotide}-\text{pair}}-E_{\text{double}-\text{counting}}\;\\ =\;\;\overset{N-1}{\underset{i=2}{\sum }}\tilde{E}({\text{Cap}}_{i-1}^{*}A_{i}{\text{Cap}}_{i+1})-\overset{N-2}{\underset{i=2}{\sum }}\tilde{E}({\text{Cap}}_{i}^{*}{\text{Cap}}_{i+1})\;\\ +\overset{N-3}{\underset{i=1}{\sum }}\overset{N}{\underset{\begin{array}{c} j=i+3\\ \left|R_{ij}\right|\leq \lambda \end{array}}{\sum }}{\left({\tilde{E}}_{ij}^{ribo}-{\tilde{E}}_{i}^{ribo}-{\tilde{E}}_{j}^{ribo}\right)}_{QM}-E_{\text{double}-\text{counting}} \end{array}$$ (1)

where $\tilde{E}$ denotes the sum of the self-energy of a fragment and the interaction energy between the fragment and its background charges. ${\text{Cap}}_{i-1}^{*}A_{i}{\text{Cap}}_{i+1}$ is the *i*th ribonucleic acid unit (*A*_*i*_) capped with a left-side cap of ${\text{Cap}}_{i-1}^{*}$ and a right-side cap of ${\text{Cap}}_{i+1}$ . The concap is fused molecular species by two neighboring ribonucleotides of ${\text{Cap}}_{i}^{*}{\text{Cap}}_{i+1}(A_{i}A_{i+1}$ ), and $\tilde{E}{\text{Cap}}_{i}^{*}{\text{Cap}}_{i+1}$ is deducted in the EE-GMFCC method to avoid the double counting of overlapping fragments. The $E_{\text{ribonucleotide}-\text{pair}}$ represents the two-body QM interaction energy of two non-neighboring ribonucleotides within a distance threshold λ, and $R_{ij}$ is the distance between the two closest atoms of *i*th and *j*th nucleotides. $\;E_{\text{double}-\text{counting}}$ is calculated for all atom pairs whose interaction energies are doubly counted in the previous terms of Eq. 1. The $E_{\text{double}-\text{counting}}$ energy is approximated by pairwise charge–charge interactions. Usually, the atomic charges taken from the Amber force field are used in the embedding electrostatic treatment of the QM calculations for all fragments and the calculation of the $E_{\text{double}-\text{counting}}$ term. The more detailed description of the total ground-state energy calculation of RNA using the EE-GMFCC method can be found in our previous work (Jin et al., 2017).

### EE-GMFCC for Excited-State Calculations of Fluorescent RNA–Aptamer Systems

#### Calculation of Excitation Energies

The EE-GMFCC method has been applied in the calculation of excitation energies of GFP in our previous work (Liu et al., 2019; Jin et al., 2020). The treatment of excited-state calculations with the EE-GMFCC method usually follows the condition of local excitation; that is, the dominant electronic reorganization that occurs in response to excitation of the system is only within a small region (Jin et al., 2020). Since the fluorophore in GFP is bonded with an amino acid as a specific residue, the excitation energy *ω* of the system could be obtained using Eqs. 2, 3, when the excitation center is in the *m*th residue.

$$\begin{array}{l} \omega =\;\overset{m+1}{\underset{i=m-1}{\sum }}\omega ({\text{Cap}}_{i-1}^{\text{*}}{\text{A}}_{i}{\text{Cap}}_{i+1})-\overset{m}{\underset{i=m-1}{\sum }}\omega ({\text{Cap}}_{i-1}^{\text{*}}{\text{Cap}}_{i+1})\\ +\overset{N}{\underset{\begin{array}{c} j=1\\ |R_{mj}|\leq \lambda_{2}\\ j\notin \left[m-2,m+2\right] \end{array}}{\sum }}\left(\omega_{mj}-\omega_{m}\right)\; \end{array}$$ (2)

where the first term is the sum of excitation energy of the (*m-*1)th, *m*th, and (*m+*1)th fragments, which can be obtained as follows:

$$\overset{m+1}{\underset{i=m-1}{\sum }}\omega ({\text{Cap}}_{i-1}^{\text{*}}{\text{A}}_{i}{\text{Cap}}_{i+1})\;=\overset{m+1}{\underset{i=m-1}{\sum }}{\tilde{E}}^{'}({\text{Cap}}_{i-1}^{\text{*}}{\text{A}}_{i}{\text{Cap}}_{i+1})-\overset{m+1}{\underset{i=m-1}{\sum }}\tilde{E}({\text{Cap}}_{i-1}^{\text{*}}{\text{A}}_{i}{\text{Cap}}_{i+1})$$ (3)

where the excitation energy *ω* for a fragment of ${\text{Cap}}_{i-1}^{*}A_{i}{\text{Cap}}_{i+1}$ (or ${\text{Cap}}_{i}^{*}{\text{Cap}}_{i+1})$ was calculated in the field of the remaining part of the protein represented by atomic charges taken from the MM force field. ${\tilde{E}}^{'}{\text{Cap}}_{i-1}^{\text{*}}A_{i}{\text{Cap}}_{i+1}\;$ is the total energy calculated at the excited state for the fragment of ${\text{Cap}}_{i-1}^{\text{*}}A_{i}{\text{Cap}}_{i+1},$ including the interactions between the excited-state wave function of ${\text{Cap}}_{i-1}^{\text{*}}A_{i}{\text{Cap}}_{i+1}\;$ and background charges of the remaining system, while $\tilde{E}{\text{Cap}}_{\text{i}-1}^{\text{*}}{\text{A}}_{\text{i}}{\text{Cap}}_{\text{i}+1}$ is the summation of the ground-state energy of ${\text{Cap}}_{i-1}^{\text{*}}A_{i}{\text{Cap}}_{i+1}$ and interaction between the fragment of ${\text{Cap}}_{i-1}^{\text{*}}A_{i}{\text{Cap}}_{i+1}$ and background charges of the remaining system. A more detailed description of the excitation energy calculation of protein using the EE-GMFCC method can be found in our previous work (Jin et al., 2020). Different from the fluorescent protein, the fluorophore binds to the Mango II RNA–aptamer through nonbonded interactions. Therefore, the excitation energy for such a system is calculated based on the EE-GMFCC method as follows,

$$\omega_{\text{EE}-\text{GMFCC}}=\omega_{\text{fluorophore}}+\omega_{\text{two}-\text{body}}=\omega_{m}+\overset{N}{\underset{\begin{array}{c} j=1\\ |R_{mj}|\leq \text{λ} \end{array}}{\sum }}\left(\omega_{mj}-\omega_{m}\right)$$ (4)

Here, $\omega_{m}$ is the calculated excitation energy for the fluorophore using the QM/MM method with the remaining part of the system as background charges. $\omega_{mj}$ represents the two-body (2B) QM excitation energy of the fluorophore *m* and *j*th ribonucleotide, which was obtained by QM calculation in the field of the rest of the system represented by the MM point charges taken from the ff99OL3 force field (Wang et al., 2000; Pérez et al., 2007; Zgarbová et al., 2011). The illustration of the 2B calculation is shown in Figure 1A.

The RNA was cut at the bond between C3 atom and O3 atom as shown in Supplementary Figure S1 of the Supporting Information. For instance, the *N*th ribonucleotide will be separated from the (*N-*1)th ribonucleotide and the (*N+*1)th ribonucleotide at the bonds of C3(*N-*1)-O3(*N-*1) and C3(*N*)-O3(*N*), where C3(*N*) and O3(*N*) represent the C3 and O3 atoms of the *N*th ribonucleotide in the RNA chain, respectively. The H atom would be utilized to saturate the dangling bond, as shown in Supplementary Figure S1. The bond length of H-O3 was set to 0.96  Å , and the bond length of C3-H was set to 1.09  Å . The QM calculations were performed using the Gaussian 16 package (Frisch et al., 2016).

#### Calculations of the TEDM and Atomic Forces

The TEDM between two states under the EE-GMFCC method could be obtained as follows:

$$\mu_{i}^{\text{EE}-\text{GMFCC}}=\mu_{i}^{m}+\overset{N}{\underset{\begin{array}{c} j=1\\ |R_{mj}|\leq {\text{λ}}_{2B} \end{array}}{\sum }}\left(\mu_{i}^{m,j}-\mu_{i}^{m}\right)$$ (5)

where $\mu_{i}^{\text{EE}-\text{GMFCC}}$ ( i=x,y,or z ) represents the calculated TEDM between two states in the *x*, *y*, or *z* direction, and $\mu_{i}^{m}$ is the TEDM of the excited state from the fragment QM calculation for the *m*th fragment (which is the fluorophore in this study) in the *x*, *y*, or *z* direction and it corresponds to the one-body (1B) term in the EE-GMFCC calculation. $\;\mu_{i}^{m,j}$ represents the calculated TEDM of the two-body fragment consisting of the fluorophore and an adjacent ribonucleotide within the distance threshold ${\text{λ}}_{2B}$ from the fluorophore. $\mu_{i}^{m,j}-\mu_{i}^{m}$ denotes the two-body QM correction for the TEDM from the interaction between the fluorophore and adjacent ribonucleotide in RNA. *N* is the total number of ribonucleotides in the RNA.

The total TEDM of the system between two states with the EE-GMFCC method could be utilized to calculate the oscillator strength as follows:

$$f^{\text{EE}-\text{GMFCC}}=\frac{2}{3}\omega^{\text{EE}-\text{GMFCC}}\left({\left|\mu_{x}^{\text{EE}-\text{GMFCC}}\right|}^{2}+{\left|\mu_{y}^{\text{EE}-\text{GMFCC}}\right|}^{2}+|{\left|\mu_{z}^{\text{EE}-\text{GMFCC}}\right|}^{2}\right)$$ (6)

where $\omega^{\text{EE}-\text{GMFCC}}$ is the calculated excitation energy using the EE-GMFCC method, and $\mu_{x}^{\text{EE}-\text{GMFCC}}$ , $\;\mu_{y}^{\text{EE}-\text{GMFCC}}\;$ , and $\mu_{z}^{\text{EE}-\text{GMFCC}}$ are calculated TEDMs in the *x*, *y*, and *z* directions, respectively.

The atomic forces of the *k*th atom in the fluorophore molecule at the excited state could also be calculated using the EE-GMFCC method by replacing the TEDM of μ in Eq. 5 with the atomic force of *F*.

$$F_{k,i}^{\text{EE}-\text{GMFCC}}=F_{k,i}^{m}+\overset{N}{\underset{\begin{array}{c} j=1\\ \left|R_{mj}\right|\leq {\text{λ}}_{2B} \end{array}}{\sum }}\left(F_{k,i}^{m,j}-F_{k,i}^{m}\right)$$ (7)

The superscripts and subscripts in Eq. 7 are similar to those in Eq. 5.

### Structure Preparation

The initial structure of the Mango-II RNA aptamer system was taken from the X-ray crystal structure (PDB id: 6C63). The generalized Amber force field (GAFF) (Wang et al., 2004) and AM1-BCC (Jakalian et al., 2000) charges were utilized to simulate the fluorophore (EKJ37) in the classical MD simulation (Walker et al., 2008). The ff99OL3 (Wang et al., 2000; Pérez et al., 2007; Zgarbová et al., 2011) force field was employed for handling the RNA. The missing hydrogen atoms were added by the LEaP module of the Amber 18 package (Case et al., 2005; Salomon-Ferrer et al., 2013; Case et al., 2018).

The residue name of the fluorophore in this study is called EKJ (in PDB id: 6C63), which consists of a thiazole orange1 (TO1) and part of the polyethylene glycol linker. The EKJ binds to the Mango II RNA with a high affinity, and the high brightness of the EKJ in the RNA allows its application in live-cell imaging and also in conventional fixed cell methodologies (Autour et al., 2018; Trachman et al., 2018). The aptamer (Mango II RNA) contains a closing RNA stem and a fluorophore-binding pocket. The high binding affinity between the fluorophore and aptamer makes it possible to discern the signal coming from the free fluorophore or the fluorophore bound to RNA, imaging small non-coding RNAs in mammalian cells (Autour et al., 2018). The structures of fluorophore, RNA stem, and binding pocket of Mango II RNA are shown in Figure 1C.

### Truncated Full-System QM Calculations

**FIGURE 2:**
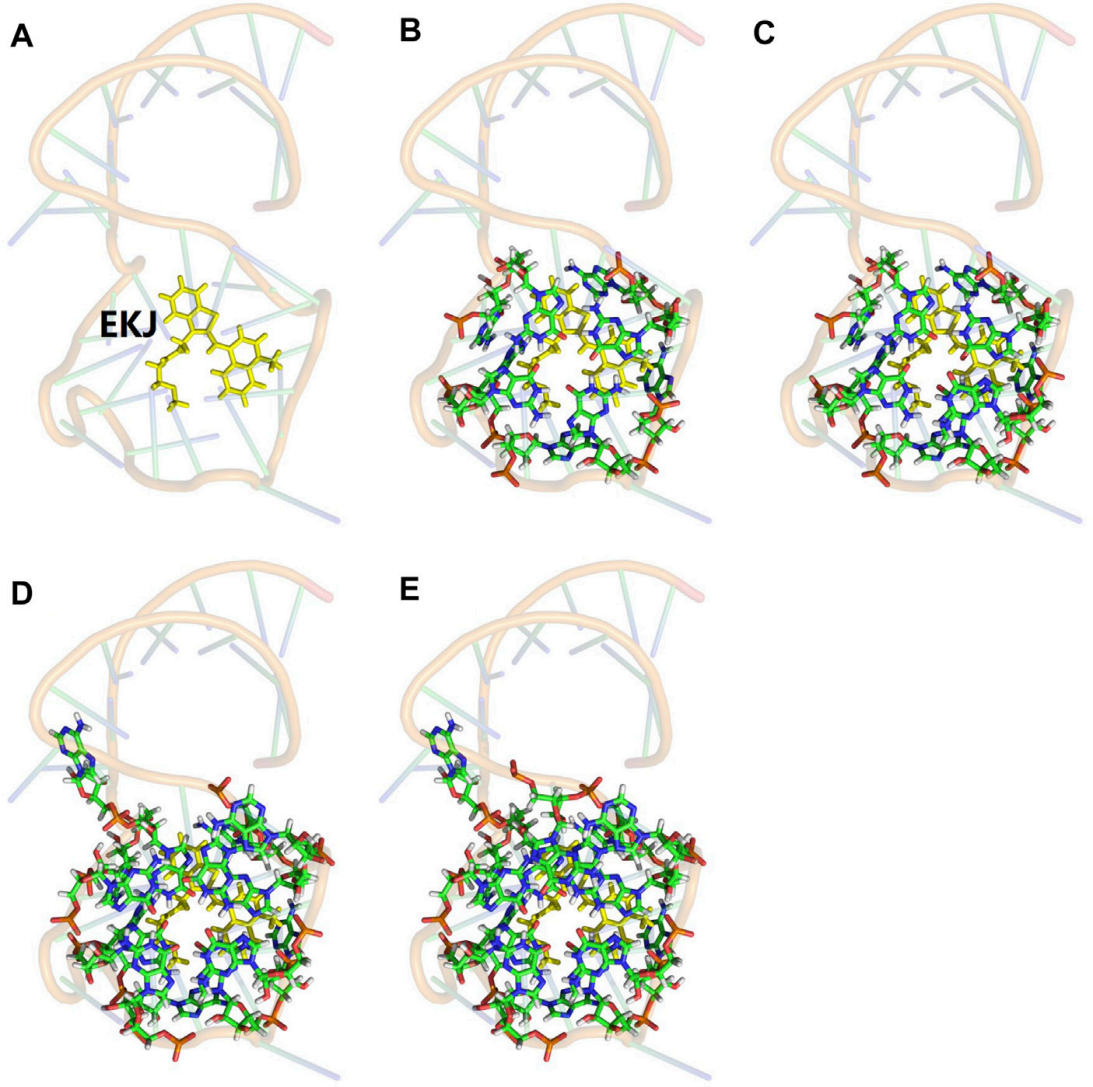
Illustration of the model system for the truncated full-system QM calculation. The QM subsystem was shown in sticks, and the MM subsystem was shown in 80% transparency backbone. **(A)** The one-body QM calculation in the EE-GMFCC method for such a system is only for fluorophore EKJ (shown in yellow). **(B)** Fullsys (4) which was constructed with a predefined distance threshold of ${\text{λ}}_{FS}\;$ = 4 Å, **(C)** fullsys (5) ( ${\text{λ}}_{FS}$ = 5 Å), **(D)** fullsys (6) ( ${\text{λ}}_{FS}$ = 6 Å), and **(E)** fullsys (7) ( ${\text{λ}}_{FS}$ = 7 Å), respectively.

The computational cost of the full-system QM calculation of Mango II RNA containing 1,243 atoms was very expensive. Considering the localization of the QM effect on the excited-state property, we constructed several smaller model systems for the Mango II RNA, which contains the fluorophore and its neighboring ribonucleotides within a predefined distance threshold ${\text{λ}}_{FS}$ (a minimum distance between any two atoms on the ribonucleotide and fluorophore, respectively), while in the QM calculation for each model system, the rest of Mango II RNA was treated by the MM model represented by the atomic point charges. Therefore, in this study the truncated full-system calculation is the traditional QM/MM calculation with the constructed central model system partitioned into the QM subsystem and the rest of the Mango II RNA partitioned into the MM subsystem. A series of model systems were constructed with different distance thresholds of ${\text{λ}}_{FS}$ = 3, 4, 5, 6, and 7 Å . The QM/MM calculations were performed on those model systems. The corresponding results of the calculated excited-state properties for different model systems were labeled as “fullsys ( ${\text{λ}}_{FS}$ )”. A detailed graphical illustration of the truncated full-system approach is shown in Figure 2.

## Results and Discussion

### Accuracy of EE-GMFCC for Excitation Energy Calculations

The accuracy of the EE-GMFCC method for prediction of the excitation energies of the RNA aptamer–fluorophore complex was investigated by comparison with the truncated full-system QM/MM calculations. In the framework of the EE-GMFCC method, a two-body QM calculation was utilized to account for the QM effect from the adjacent ribonucleotides on the calculations of excited-state properties of the fluorescent aptamer. A predefined distance threshold ( ${\text{λ}}_{2B}$ ) was used in the 2B calculations to achieve the balance between the attained accuracy and computational efficiency. The convergence of EE-GMFCC calculations as a function of ${\text{λ}}_{2B}$ was first tested for both the TD-HF and TD-DFT methods by comparison with the truncated full-system calculations. ${\text{λ}}_{FS}$ was set to 3, 4, 5, 6, and 7 Å, respectively, to test the impact of the chemical environment on calculations of the excitation energies. The truncated full-system calculations demonstrated that the difference of the obtained excitation energies between fullsys (6) and fullsys (7) was merely 0.001 eV, as shown in Supplementary Figure S2 of the Supporting Information. Therefore, the system constructed with ${\text{λ}}_{FS}$ = 7 Å was used as the benchmark system for investigating the ${\text{λ}}_{2B}$ dependence of EE-GMFCC calculations. Furthermore, the results in Supplementary Figure S2 also show that the calculated excitation energy of fullsys ( ${\text{λ}}_{FS}$ = 4 Å) was very close to that of fullsys ( ${\text{λ}}_{FS}$ = 7 Å) at both TD-HF/6-31G* and TD-ωB97X/6-31G* levels. This indicates that, to some extent, the constructed “full system” with ${\text{λ}}_{FS}$ of 4 Å can sometimes be used as a candidate system for some benchmark studies of the EE-GMFCC method.

**FIGURE 3:**
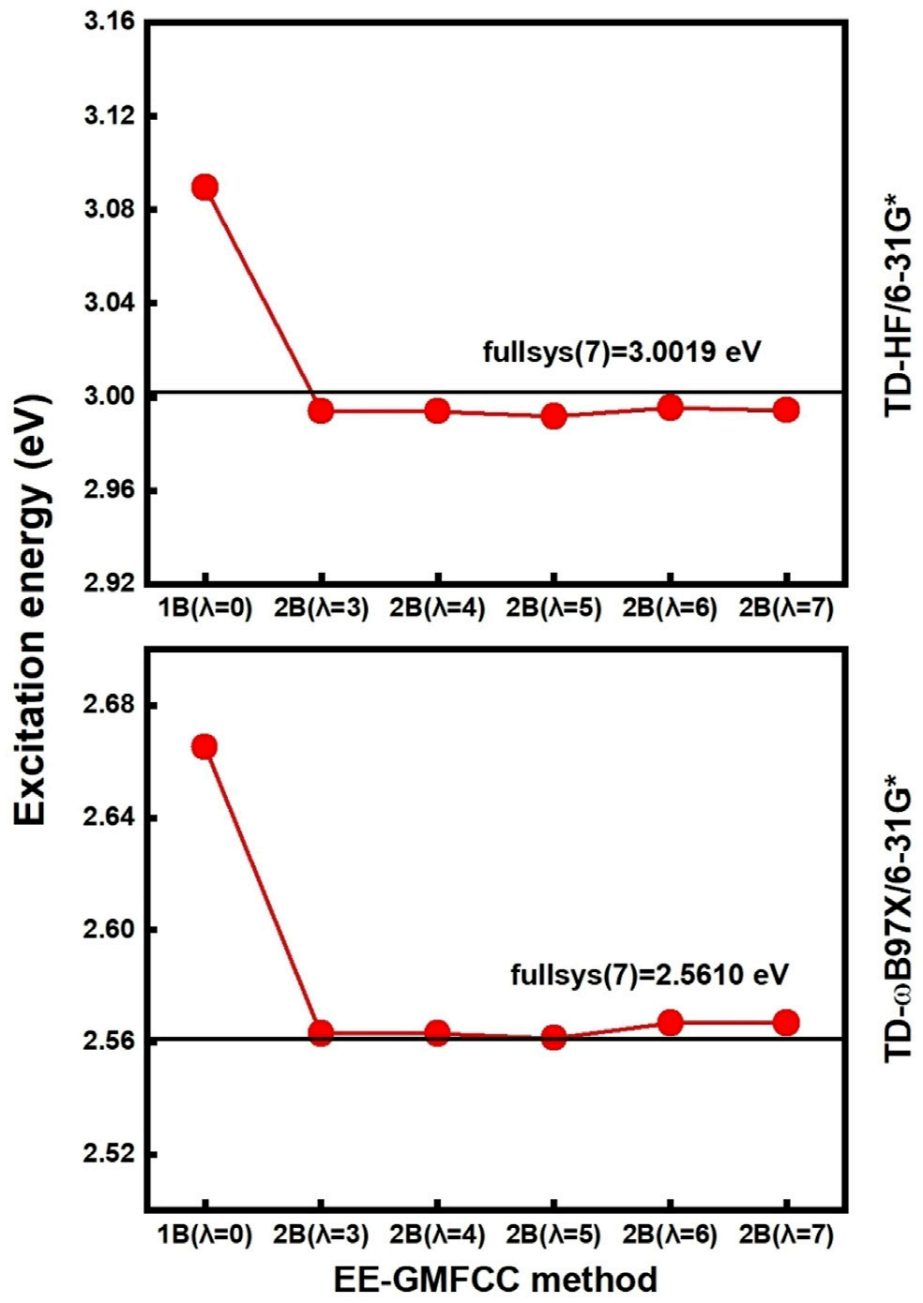
Calculated excitation energies (red line) using EE-GMFCC as a function of the distance threshold of ${\text{λ}}_{2B}$ at the TD-HF/6-31G* and TD-ωB97X/6-31G* levels, respectively. The calculated excitation energies from the model system of fullsys (7) constructed using ${\text{λ}}_{FS}$ = 7 Å are regarded as the reference (black line).

The calculated excitation energy using truncated full-system calculation of the system would be set as the reference. The results of the excitation energies calculated by EE-GMFCC with different ${\text{λ}}_{2B}$ are shown in Supplementary Table S1 of the Supporting Information. It can be seen that the results of EE-GMFCC calculations are close to convergence when ${\text{λ}}_{2B}$ is set to 4 Å (see Figure 3), and the result is also close to that of the full-system calculation ( ${\text{λ}}_{FS}$ = 7 Å) with the absolute error within 0.008 eV. Therefore, we conclude that the distance threshold of 4 Å is sufficient for the two-body QM calculation of the excitation energies using the EE-GMFCC method.

### Calculation of TEDM and Atomic Forces Using EE-GMFCC

**FIGURE 4:**
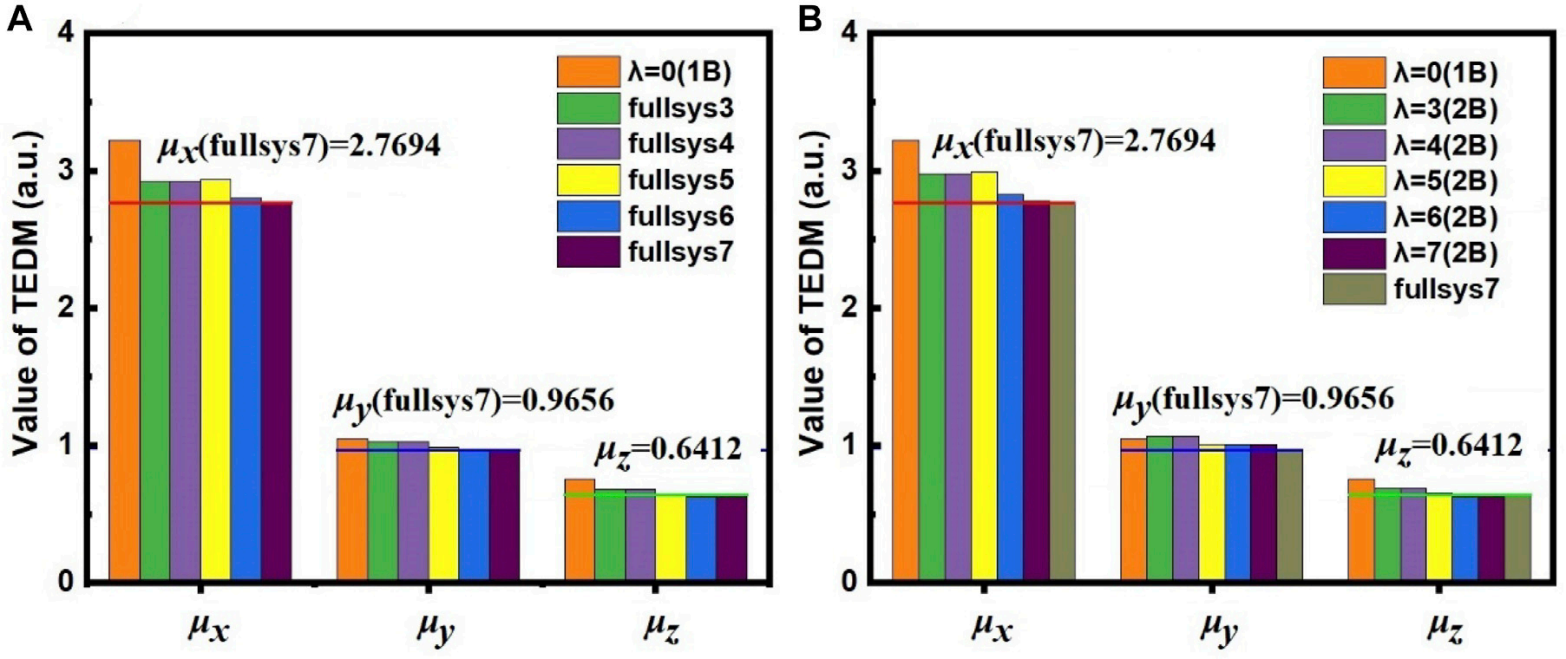
The calculated *μ*_*x*_, *μ*_*y*_, and *μ*_*z*_ of TEDM with **(A)** full-system calculations and **(B)** the EE-MFCC method for different model systems constructed using various $\lambda_{\text{FS}}$ at the TD-HF/6-31G* level. The red (*μ*_*x*_), blue (*μ*_*y*_), and green (*μ*_*z*_) lines denote the corresponding values calculated on the truncated full system of fullsys (7).

In addition to excitation energies, EE-GMFCC can also be utilized in the calculations of other excited-state properties, including TEDM (Tanabe et al., 1965; Verma et al., 2020) and atomic forces (Weisenhorn et al., 1989; Sarid et al., 1991). The calculated TEDMs for different model systems constructed with different ${\text{λ}}_{FS}$ of 4, 5, 6, and 7 Å using the EE-GMFCC method are shown in Figure 4B, and the ${\text{λ}}_{2B}$ for two-body QM calculations with the EE-GMFCC method was also set to the same value as ${\text{λ}}_{FS}$ . For comparison, the results of the TEDM calculated using truncated full-system calculation are also shown in Figure 4.

One can see from Figure 4A that the calculated TEDM of the system of 1B ( ${\text{λ}}_{FS}$ = 0 Å ) shows a relatively larger deviation from that of fullsys (3), especially for *µ*_*x*_, which indicates that incorporating the local QM effects from the environment is crucial for accurate prediction of the TEDM. For comparison, the results of the calculated TEDM and the oscillator strength at the TD-HF/6-31G*^13^ level for different model systems using the truncated full-system and EE-GMFCC calculations are also shown in Table 1 (the corresponding results calculated at the TD-ωB97X/6-31G* (Chai and Head-Gordon, 2008) level are shown in Supplementary Table S2 of the Supporting Information). It can be seen that the results of the EE-GMFCC method are in good agreement with the full-system calculations for all excited-state properties of different model systems.

**TABLE 1:** Transition electric dipole moment (TEDM) calculated by EE-GMFCC and corresponding full-system calculations at the TD-HF/6-31G* level for different model systems with different distance thresholds $\lambda_{FS}$ . *μ*_*x*_, *μ*_*y*_, and *μ*_*z*_ are the calculated TEDM values in *x*, *y*, and *z* directions, respectively. *ω* and *f* are the excitation energy and the corresponding oscillator strength, respectively. Here, the $\lambda_{2B}$ employed in EE-GMFCC calculations for different model system was set to the same value as the $\lambda_{FS}$ .

$\lambda_{FS}$ (Å)		*μ*_x_ (a.u.)	*μ*_y_ (a.u.)	*μ*_z_ (a.u.)	ω (eV)	*f*
-	1B	3.2193	1.0 4 87	0.7567	3.0896	0.9111
4	2B	2.9735	1.0657	0.6880	2.9938	0.7666
	Fullsys (4)	2.9222	1.0278	0.6810	2.9954	0.7382
5	2B	2.9887	1.0059	0.6550	2.9917	0.7603
	Fullsys (5)	2.9392	0.9858	0.6411	2.9966	0.7358
6	2B	2.8269	1.0060	0.6267	2.9954	0.6896
	Fullsys (6)	2.8015	0.9762	0.6288	3.0018	0.6764
7	2B	2.7828	1.0056	0.6438	2.9943	0.6727
	Fullsys (7)	2.7694	0.9656	0.6412	3.0019	0.6629

**FIGURE 5:**
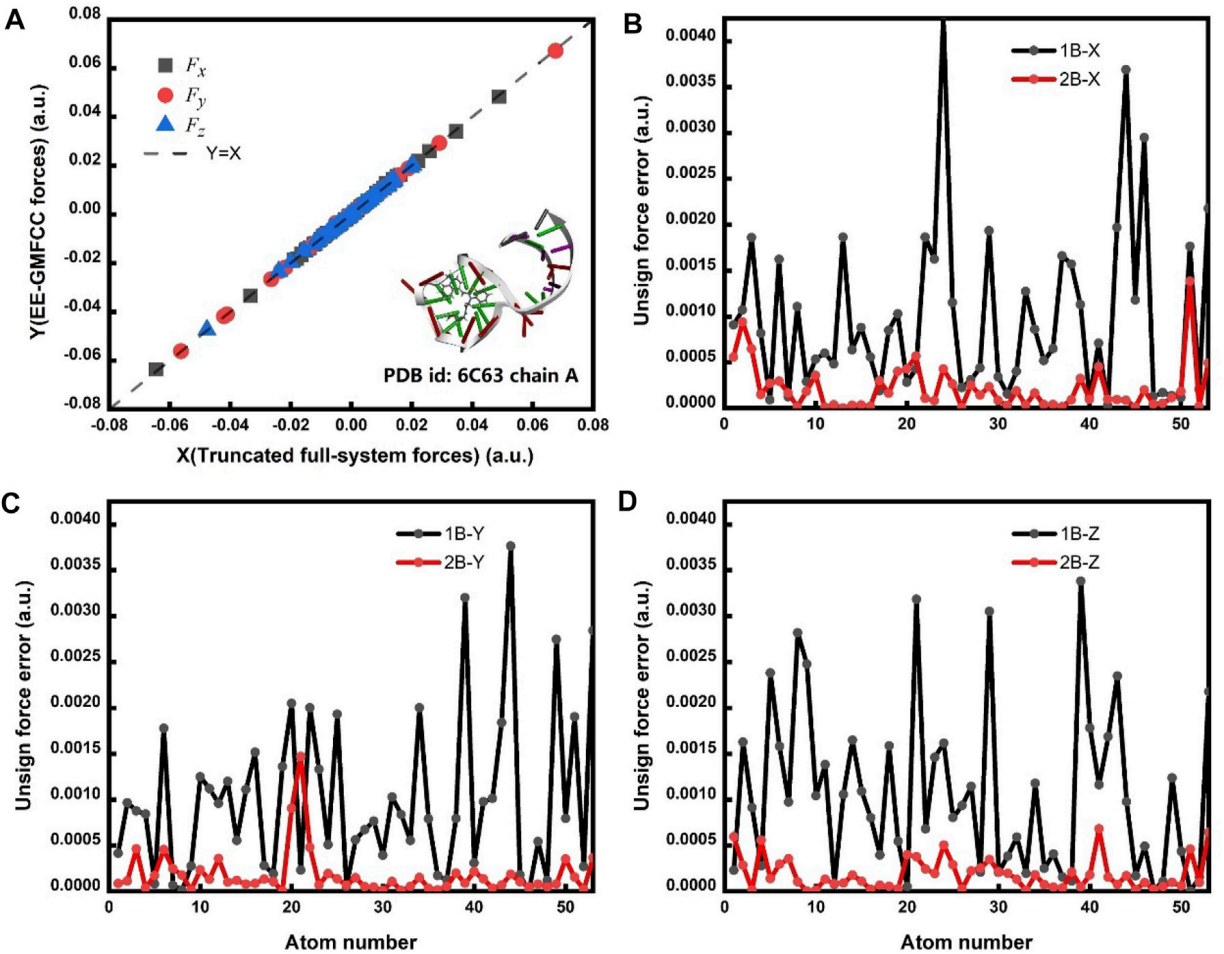
**(A)** Correlation of the calculated atomic forces between the EE-GMFCC method and truncated full-system calculations. *F*_*x*_, *F*_*y*_, and *F*_*z*_ are the calculated atomic forces for all atoms of the fluorophore in *x*, *y*, and *z* directions, respectively. The calculations were performed at the TD-HF/6-31G* level. The dashed line is the strict correlation curve. The mean unsigned error (MUE) between the EE-GMFCC and the truncated full-system method is 0.00022 hartree/bohr. **(B)** The unsigned error for *F*_*x*_ between the EE-GMFCC method and truncated full-system calculations under the one-body (1B) and two-body (2B) treatment. **(C)** Similar to panel b but for *F*_*y*_. **(D)** Similar to panel b but for *F*_*z*_.

The correlation of the calculated atomic forces for the fluorophore molecule between the EE-GMFCC and full-system calculations is shown in Figure 5A. The results demonstrate that the EE-GMFCC method could reproduce well the corresponding atomic forces from full-system calculations with a mean unsigned error lower than 0.001 hartree/bohr. The absolute errors of the calculated atomic forces in *x*, *y*, and *z* directions using EE-GMFCC-1B and EE-GMFCC-2B with reference to full-system calculations are shown in Figures 5B–D, respectively. The results show that EE-GMFCC-2B could provide more accurate results for atomic forces than the EE-GFMCC-1B treatment, indicating that accounting for the QM effects from the local chemical environment is essential for calculations of atomic forces.

### Computational Efficiency of EE-GMFCC

**FIGURE 6:**
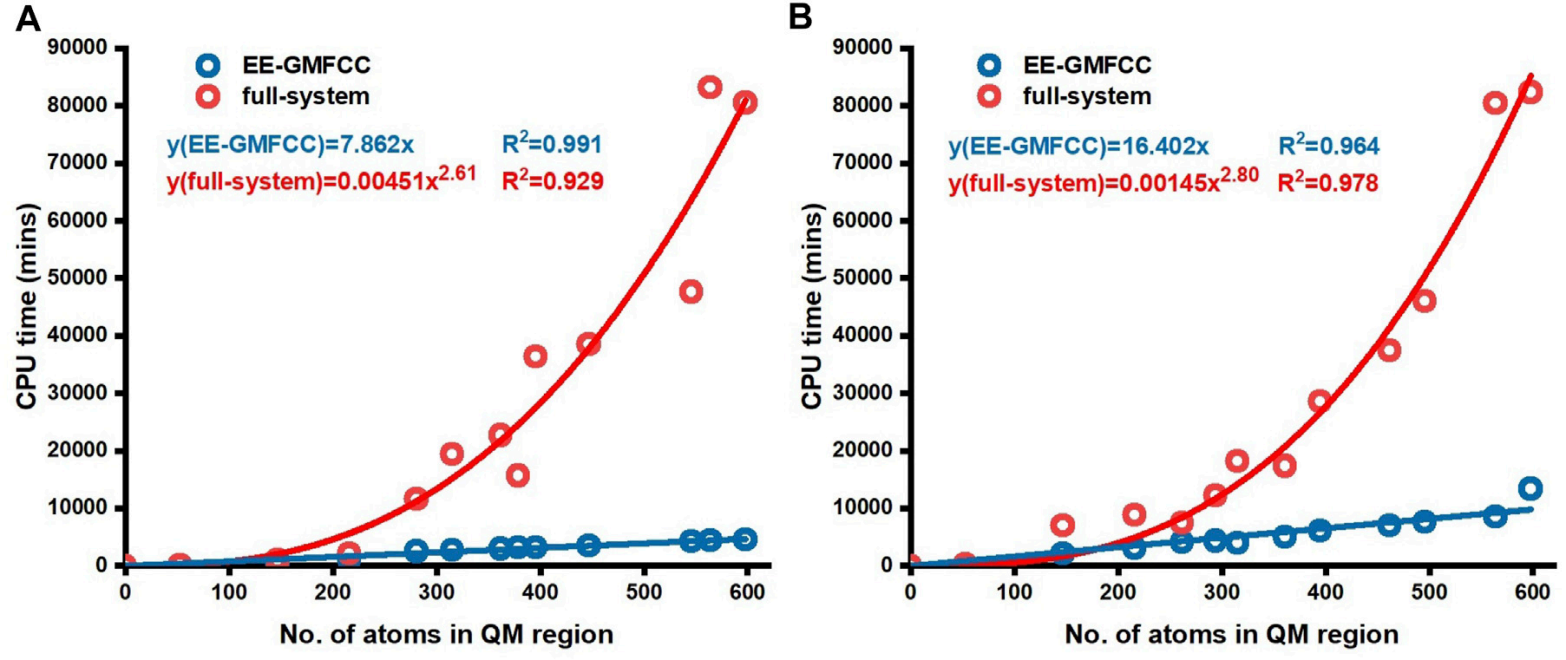
CPU time for conventional full-system and EE-GMFCC calculations as a function of the number of atoms of the truncated model systems. The calculations were performed **(A)** at the TD-HF/6-31G* level and **(B)** at the TD-ωB97X/6-31G* level, respectively.

Figure 6 shows the comparison of the CPU time for excitation energy calculations of different Mango-II RNA aptamer systems on the Intel Xeon Gold 6,130 2.1-GHz processor with the full-system calculations and EE-GMFCC approach at the TD-HF/6-31G* and TD-ωB97X/6-31G* levels, respectively. As expected, the computational scale of the EE-GMFCC approach shows O(*N*) as a function of the number of atoms in the system, while the computational cost for the traditional full-system TD-HF and TD-DFT calculations exhibits O ( $N^{2.61}$ ) and O ( $N^{2.80}$ ), respectively. The obtained regression equation for the full-system calculations at the TD-HF/6-31G* level is $y=0.00451x^{2.61}$ . In contrast, it is *y* = 7.862*x* for EE-GMFCC. As mentioned above, the calculated excited-state properties could reach convergence when ${\text{λ}}_{\text{FS}}$ is set to 4 Å. For the model system constructed with the distance threshold of $\lambda_{\text{FS}}$ = 4 Å the full-system calculation took 22,729 min of CPU time at the TD-HF/6-31G* level. In contrast, the EE-GMFCC method took only 2,957 min. The scale of the EE-GMFCC method at TD-ωB97X/6-31G* has a similar trend as compared to that at the TD-HF/6-31G* level (see Figure 6B).

### Prediction of the Relative Excitation Energies Using EE-GMFCC

The performance of the EE-GMFCC method on prediction of the relative excitation energies of different configurations was also investigated. A 100-ns classical MD simulation was first carried out on the Mango II RNA in explicit water solvent, and then excitation energy calculations were performed with both the EE-GMFCC and truncated full-system approaches on 10 different configurations of the Mango II RNA system extracted from the MD simulation trajectory.

**FIGURE 7:**
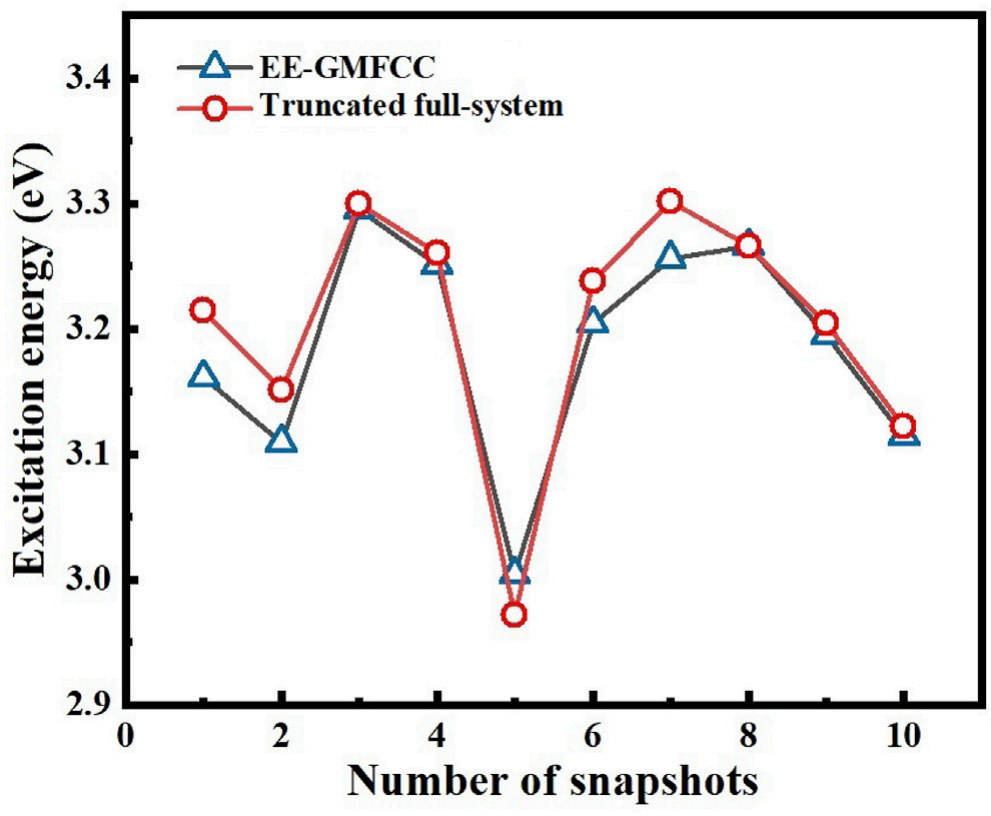
Comparison of the calculated excitation energies for 10 different configurations of the fluorescent RNA–aptamer (PDB id: 6C63) system generated from the 100-ns MD simulation between the EE-GMFCC-2B approach and the truncated full-system calculations at the TD-HF/6-31G* level. $\lambda_{2\text{B}}$ was set to 4 Å for the two-body QM calculations in EE-GMFCC. $\lambda_{\text{FS}}$ was set to 4 Å for the truncated full-system calculations.

The calculated excitation energies at the TD-HF/6-31G* level are shown in Table 2 and Figure 7. It can be seen that the predicted relative excitation energies by EE-GMFCC-2B show good agreement with those from full-system calculations with the mean unsigned deviation (MUD) of 0.02 eV. The calculated relative excitation energies of the 10 different configurations with EE-GMFCC-1B are also shown in Table 2, which shows larger errors compared to EE-GMFCC-2B with reference to full-system calculations (MUD = 0.08 eV). The results demonstrate that QM treatment of the RNA local chemical environment is essential for accurate calculation of both absolute and relative excited-state properties. The excitation energies calculated at the TD-ωB97X/6-31G* level are shown in Supplementary Table S3 of the Supporting Information.

**TABLE 2:** Predicted excitation energies for 10 different configurations generated from the 100-ns classical MD simulation for the fluorescent RNA–aptamer (PDB id: 6C63) system using the EE-GMFCC and truncated full-system calculations at the TD-HF/6-31G* level. The model systems were constructed with $\lambda_{\text{FS}}\;$ = 4 Å for the 10 different configurations. A distance threshold $\lambda_{2\text{B}}$ = 4 Å was employed for two-body QM calculations in EE-GMFCC.

Snapshots	1B (eV) ( $\lambda_{2\text{B}}$ = 4Å)	2B (eV) ( $\lambda_{\text{FS}}$ = 4Å)	Fullsys (4)	Deviation 1^a^ (eV)	Deviation 2^b^ (eV)
1	3.334	3.162	3.215	0.119	−0.053
2	3.256	3.110	3.151	0.105	−0.041
3	3.397	3.295	3.300	0.097	−0.005
4	3.349	3.251	3.261	0.088	−0.010
5	3.070	3.005	2.972	0.098	0.033
6	3.307	3.204	3.238	0.069	−0.034
7	3.404	3.256	3.302	0.102	−0.046
8	3.329	3.266	3.266	0.063	0.000
9	3.218	3.195	3.204	0.014	−0.009
10	3.174	3.115	3.123	0.051	−0.008
MUD^c^	-	-	-	0.081	0.024

a Deviation of excitation energy between EE-GMFCC(1B) and truncated full-system calculations.

b Deviation of excitation energy between EE-GMFCC(2B) and truncated full-system calculations.

c MUD, denotes the mean unsigned deviation.

### Ribonucleotide-Based Decomposition of Excitation Energies

Investigation of the ribonucleotide-based decomposition of the excitation energy around the fluorescent molecule (Shen et al., 2021) is essential for finding the so-called hotspots and attendant rational design of the fluorescent RNA–aptamer complex using the point mutation technology. Herein, the fragmentation QM method was utilized to decompose the contribution of each ribonucleotide to the excitation energy. Since the electrostatic embedding treatment in the EE-GMFCC method would incorporate many-body effects, which obscures the individual contribution, the GMFCC scheme (without the electrostatic embedding field) was thus employed. The fragmentation treatment of the GMFCC method for RNA (or protein) systems is the same as that of the EE-GMFCC approach. However, in QM calculation of each fragment with the GMFCC scheme, the background charges were not introduced as compared to the EE-GMFCC method. The influence of each ribonucleotide around the fluorophore molecule on the calculated excitation energy was predicted by GMFCC. The results of 10 different configurations extracted from the 100-ns MD simulation were utilized to approximately represent the ensemble-averaged value due to the expensive computational cost. The excitation energies were calculated at the TD-HF/6-31G* and TD-ωB97X/6-31G* levels, respectively.

**FIGURE 8:**
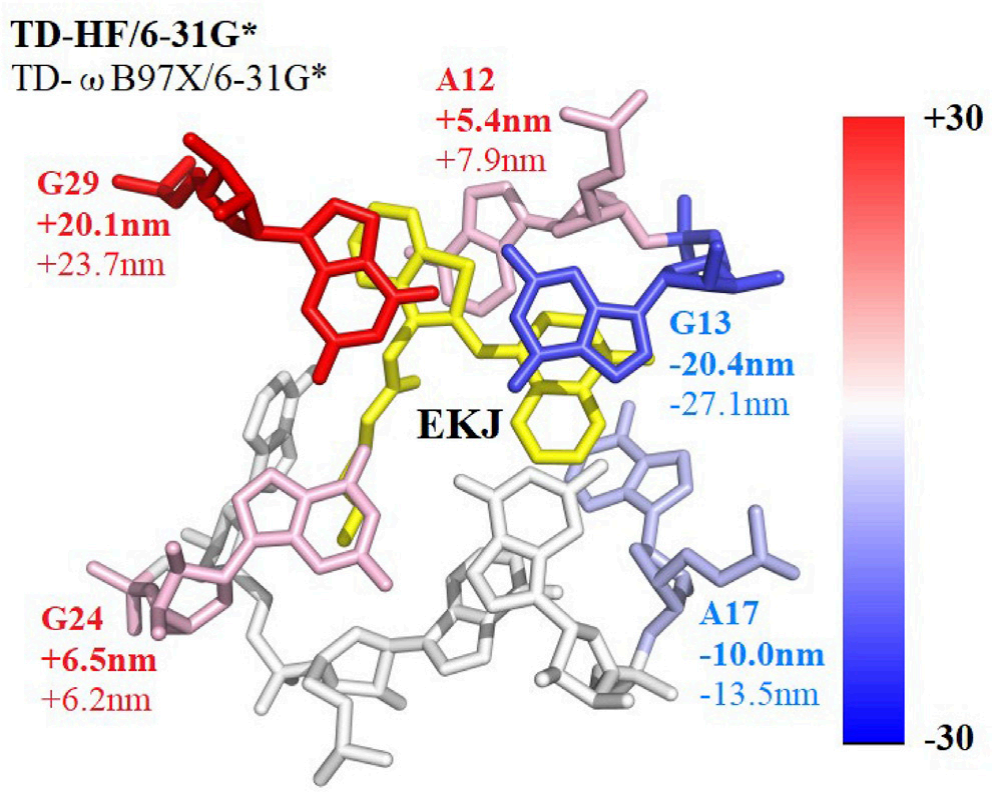
Decomposed excitation energy contributions of some ribonucleotides close to the fluorophore molecule using the GMFCC method. The results are the average values calculated on 10 snapshots extracted from the 100-ns MD simulation every 10 ns. The contributions were converted into wavelengths and presented by the color between blue and red. Ribonucleotides with positive 2B QM corrections have blue shifts of the absorption spectrum and are colored in blue, while ribonucleotides with negative 2B QM corrections have red shifts and are colored in red. EKJ is the fluorophore in this RNA system. The wavelength contribution of each ribonucleotide with bold comes from TD-HF/6-31G* calculations, while those without bold come from TD-ωB97X/6-31G* results, respectively.

The decomposition of the excitation energy of the fluorescent RNA–aptamer (PDB id: 6C63) is shown in Table 3 and Figure 8. One can see that the G13, A17, and G29 ribonucleotides contribute mostly to the calculated excitation energy. However, the G13 and A17 ribonucleotides give blue-shift contributions to the excitation spectrum, and the G29 ribonucleotide gives a red-shift contribution to the excitation spectrum. As shown in Figure 8, the spatial positions of those three ribonucleotides are close to the fluorophore molecule, and the G13 and G29 ribonucleotides locate at the right and left sides of the fluorophore molecule, respectively. The opposite effects of the two ribonucleotides (G13 and G29) on the excitation energy indicate the importance of relative spatial location.

**TABLE 3:** Contributions of the ribonucleotides in close contact with the fluorophore to the calculated excitation energy predicted by the GMFCC approach, based on 10 snapshots extracted from the 100-ns MD simulation. The calculations were performed at the TD-HF/6-31G* and TD-ωB97X/6-31G* levels, respectively. “Ex” is the calculated excitation energy (in eV) for the fluorophore molecule of EKJ37 or the two-body (2B) molecular species consisting of one ribonucleotide in RNA and EKJ37 (shown in ribonucleotide name in the table). “ΔEx” represents the excitation energy difference between the two-body (2B) molecular fragment and EKJ37, and ΔWL represents the wavelength difference (in nm) converted from ΔEx.

	Name	Ex (eV)	ΔEx (meV)	ΔWL (nm)
TD-HF/6-31g*	EKJ37	3.309	0	0.0
	A12	3.261	−48	5.4
	G13	3.499	190	−20.4
	A17	3.399	90	−10.0
	G18	3.319	10	−1.2
	A22	3.328	19	−2.2
	A23	3.281	−28	3.2
	G24	3.252	−57	6.5
	G29	3.141	−68	20.1
TD-ωB97X/6-31G*	**Name**	**Ex (eV)**	**ΔEx (meV)**	**ΔWL(nm)**
	EKJ37^a^	2.821	0	0.0
	A12	2.771	−50	7.9
	G13	3.005	184	−27.1
	A17	2.910	89	−13.5
	G18	2.823	2	−0.4
	A22	2.841	20	−3.2
	A23	2.803	−18	2.8
	G24	2.781	−40	6.2
		G29	2.677	−144	23.7

Previous theoretical and experimental studies (Park and Rhee, 2016; Hagras and Glover, 2018; Langeland et al., 2018; Jin et al., 2020; Romei et al., 2020) on the GFP have emphasized the significant influence of the electrostatic effect from the protein environment on the fluorescence of the chromophore. Here, the two-body fragments constructed using the GMFCC method were utilized to further investigate the possible physical origins of the influence of the environment. The excitation energies for a series of two-body fragments were calculated using full QM and QM/MM methods, respectively. The rest of the RNA was excluded in all of those calculations to avoid the ambiguity caused by multiple interactions from the complex environment.

The results of QM/MM calculations are usually affected by the MM parameters for mimicking the atomic point charges. For more accurately reproducing the classical electrostatic effect of the adjacent ribonucleotide on the excited-state properties of the fluorescent molecule in the full 2-body QM calculation, the excited-state calculations were first performed with the full-system QM method (the given two-body fragment including the corresponding adjacent ribonucleotide and the fluorescent molecule was treated by the QM method), and then the obtained ESP charges were utilized in the QM/MM calculations to serve as the background charges (the results were labeled as QM/ESP). For investigating the parameter dependence of the QM/MM calculations, the excited-state calculations were also performed with the adjacent ribonucleotide represented by the ff99OL3 force field.

As shown in Supplementary Table S5 of the Supporting Information, the results of the QM/ESP method are different from those of the QM/OL3 calculations, and the largest deviation between the two methods is up to 0.042 eV (the fluorophore-A15 fragment of the 6UP0 chain-C system), indicating the significant MM parameter dependence of the excited-state QM/MM calculations on the fluorescent RNA system. Since the ESP charges used in the excited-state QM/ESP calculations were taken from the full QM calculations, they could be taken as the good representation of the classical electrostatic interactions. While a significant difference can be found between the QM and QM/ESP methods, the deviations between the two methods are up to 0.094 eV for the fluorophore-G14 fragment, and 0.093 eV for the fluorophore-A15 fragment of the 6UP0 chain-C system, respectively. However, both treatments (ESP and ff99OL3 representations for mimicking the MM point charges) for the QM/MM calculations could give the correct direction of the change of the calculated excited-state energies with reference to the full QM calculations for most of the two-body fragments except the fluorophore-G10 fragment of the 6UP0 chain-C system and the fluorophore-A12 fragment of the 6C63 system with small two-body effects, indicating the important influence of the classical electrostatic interactions on the calculation of the excited-state energies.

Accordingly, the opposite effects (the blue and red shifts) of the same kind of ribonucleotides (G13 and G29, A12, and A17) on the excitation energy (see Figure 8) might be explained by the fact that the fluorescent molecule experiences the electric field in the opposite directions exerted by those adjacent ribonucleotides, which is consistent with the previous study by Park and Rhee (Park and Rhee, 2016). Overall, the results demonstrate that both the classical Coulomb interaction and the quantum exchange effects play significant roles in the calculations of the excited-state energies for the RNA–aptamer systems.

### The Application of the EE-GMFCC Method for Other Fluorescent RNA–Aptamer Systems

**FIGURE 9:**
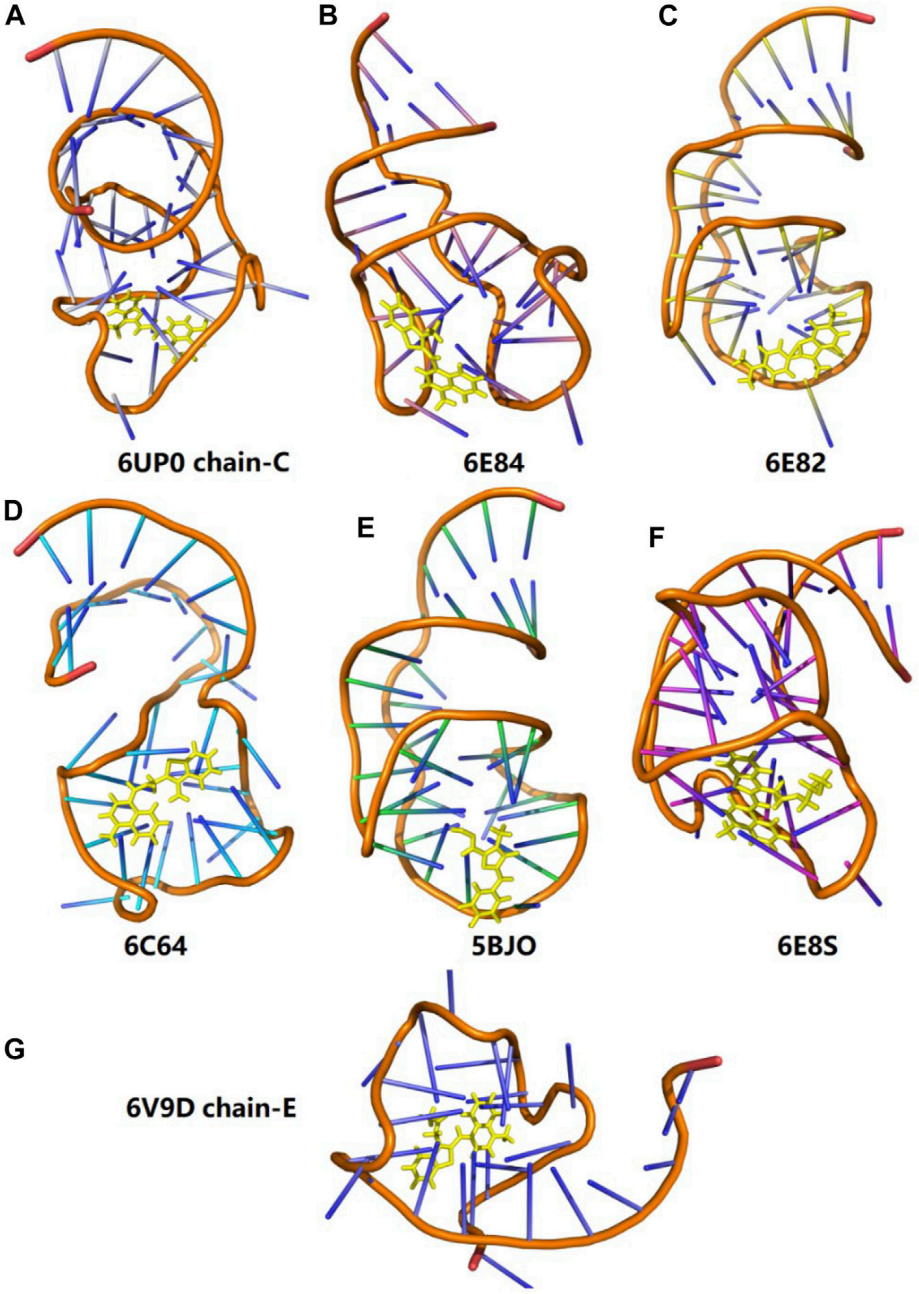
Three-dimensional structures of seven different fluorescent RNA–aptamer systems. The fluorophore is shown in the yellow stick model.

**FIGURE 10:**
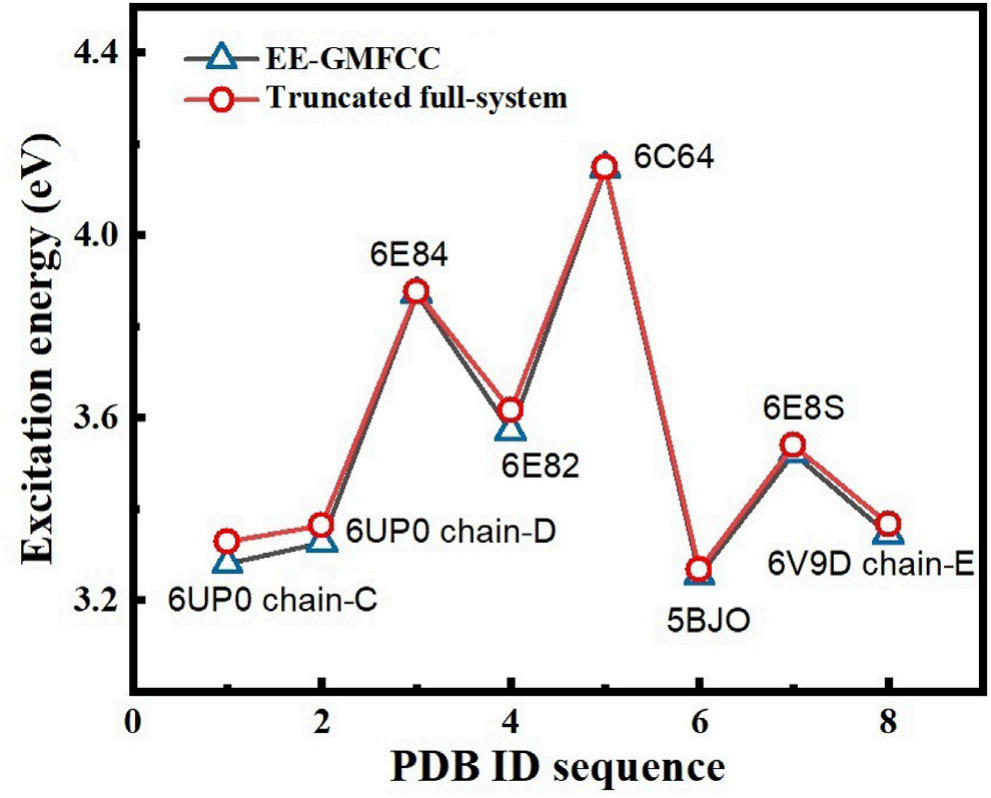
Comparison of the excitation energies of eight different fluorescent RNA systems between the EE-GMFCC approach and the truncated full-system calculations at the TD-HF/6-31G* level. The distance threshold $\lambda_{2\text{B}}$ was set to 4.0 Å for the two-body QM calculations in the EE-GMFCC method. $\lambda_{\text{FS}}$ was set to 4 Å for the truncated full-system approach.

In order to test the performance of the EE-GMFCC method on different fluorescent RNA–aptamer systems, the calculations of the excitation energies for seven other fluorescent RNA–aptamer systems taken from the PDB (shown in Figure 9) were performed using the EE-GMFCC and truncated full-system methods, respectively. The calculated excitation energies using the EE-GMFCC and full-system calculations are shown in Table 4 and Figure 10. One can see from Table 4 that EE-GMFCC-2B can give an accurate excitation energy prediction for all the fluorescent RNA–aptamer systems at the TD-HF/6-31G* level, as compared to the truncated full-system calculations, with the MUD of 0.024 eV, which demonstrates that the EE-GMFCC method is a general approach for an accurate prediction of the excited-state properties of the fluorescent RNA–aptamer systems. In contrast, the MUD of the EE-GMFCC-1B results is 0.145 eV with reference to the full-system calculations, and the absolute deviations between the EE-GMFCC-1B and full-system calculations could reach up to 0.228, 0.245, and 0.218 eV for the systems of 6UP0 chain-C, 6UP0 chain-D, and 6E8S, respectively, indicating the importance of the QM treatment of the local chemical environment in the calculation of the excited-state properties. Therefore, the EE-GMFCC-2B method is recommended to be employed in the study requiring a highly accurate prediction of excitation energies, while the EE-GMFCC-1B approach can be applied in qualitative or semiquantitative studies for efficiency. The calculated excitation energy results at the TD-ωB97X/6-31G* level are shown in Supplementary Table S4 of the Supporting Information.

**TABLE 4:** Comparison of the calculated excitation energies for a series of fluorescent RNA–aptamer systems between the EE-GMFCC ( $\lambda_{2\text{B}}$ = 4 Å) and truncated full-system calculations at the TD-HF/6-31G* level. The model systems constructed with $\lambda_{\text{FS}}$ = 4 Å for all fluorescent RNA–aptamer systems are utilized here.

PDB ID	1B (eV) (eV)	2B (eV) (λ = 4Å)	Fullsys (4Å)	Deviation 1^a^ (eV)	Deviation 2^b^ (eV)
6UP0 chain-C	3.557	3.280	3.329	0.228	−0.049
6UP0 chain-D	3.610	3.327	3.365	0.245	−0.038
6E84	3.809	3.872	3.877	−0.068	−0.005
6E82	3.684	3.571	3.617	0.067	−0.046
6C64	4.249	4.146	4.149	0.100	−0.003
5BJO	3.381	3.254	3.266	0.115	−0.012
6E8S	3.759	3.522	3.541	0.218	−0.019
6V9D chain-E	3.489	3.345	3.367	0.122	-0.022
MUD^c^	-	-	-	0.145	0.024

a Deviation of the excitation energies between EE-GMFCC(1B) and truncated full-system calculations.

b Deviation of the excitation energies between EE-GMFCC(2B) and truncated full-system calculations.

c MUD, denotes the mean unsigned deviation.

For further illustrating the performance of the EE-GMFCC-2B method on predicting the relative excitation energies between different configurations and different systems of the RNA–aptamer complex, the correlations of calculated excitation energies between the EE-GMFCC (1B and 2B) method and truncated full-system calculations at the TD-HF/6-31G* level are plotted in Supplementary Figure S3 of the Supporting Information, for different configurations of the 6C63 system (Supplementary Figure S3a) and different fluorophore RNA–aptamer systems (Supplementary Figure S3b), respectively. The results show that the EE-GMFCC-2B method gives a better correlation with the truncated full-system calculations than the EE-GMFCC-1B method. The correlation coefficients (*R^2^* (Dolgosheina et al., 2014)) of the EE-GMFCC-2B method are 0.937 for different configurations of 6C63, and 0.998 for different fluorophore RNA–aptamer systems, respectively, while the correlation coefficients (*R^2^* (Dolgosheina et al., 2014)) of the EE-GMFCC-1B method are only 0.909 and 0.878, respectively. The results demonstrate that the EE-GMFCC-2B method is capable of providing a better description of the relative excitation energies for the RNA–aptamer system than the EE-GMFCC-1B method.

## Conclusion

In this study, the electrostatically embedded generalized molecular fractionation with conjugate caps (EE-GMFCC) method was applied to calculations of the excited-state properties of the fluorescent RNA aptamer systems. The two-body fragment QM calculations were utilized to account for the QM effect from the local RNA chemical environment on the excited-state properties of the fluorescent molecule. The benchmark study on the Mango-II RNA aptamer system demonstrated that EE-GMFCC could give good agreement with traditional full-system QM calculations of the absolute and relative excitation energies, and the 4 Å distance threshold for the two-body QM calculations could strike a good balance between the attained accuracy and the computational expense incurred for the EE-GMFCC method. Furthermore, the EE-GMFCC method could provide an accurate prediction of other excited-state properties, namely, TEDM and atomic forces. This work demonstrated that incorporating the QM effects of a local RNA chemical environment was essential for an accurate prediction of the excited-state properties of the fluorescent molecule in RNAs, and hundreds of atoms were usually required to be treated with electronic structure theories. It is challenging for the traditional full-system QM calculation to handle such large systems due to the expensive computational cost. In contrast, the computational cost of the EE-GMFCC method is linear-scaling with a low prefactor, and thus the EE-GMFCC approach is computationally efficient, which could be applied for tackling the macromolecular systems. The applications of the EE-GMFCC method in calculations of the excitation energies for different fluorescent RNA aptamer systems demonstrate that the EE-GMFCC is a general approach for the excited-state property calculations of large complex molecular systems.

### Supporting Information

Illustration of the EE-GMFCC fragmentation scheme; Calculated excitation energies as a function of the distance threshold; Calculated TEDM at the TD-ωB97X/6-31G* level for different model systems using the truncated full-system and EE-GMFCC method; The relative excitation energies for different configurations of the RNA system (pdb id: 6C63) predicted by the EE-GMFCC method and truncated full-system calculations at the TD-ωB97X/6-31G* level; The relative excitation energies for different RNA systems predicted by the EE-GMFCC method and truncated full-system calculations at the TD-ωB97X/6-31G* level.

## Data Availability

The raw data supporting the conclusions of this article will be made available by the authors, without undue reservation.
