# Supplementary material for: Fragment-Based Quantum Mechanical Calculation of Excited-State Properties of Fluorescent RNAs
Source: Front Chem. 2021 Dec 22;9:801062. doi: 10.3389/fchem.2021.801062 (PMC8727457; doi:10.3389/fchem.2021.801062)
Supplement: Supplementary file 1 [file DataSheet1.PDF]

The Supporting Information for

Fragment-based Quantum Mechanical Calculation of Excited-State Properties of fluorescent RNAs

Chenfei Shen[1], Xianwei Wang[2] and Xiao He[1,3]**

[1]*Shanghai Engineering Research Center of Molecular Therapeutics and New Drug Development, School of Chemistry and Molecular Engineering, East China Normal University, Shanghai, 200062, China*

[2]*College of Science, Zhejiang University of Technology, Hangzhou 310023, Zhejiang, China*

[3]*New York University-East China Normal University Center for Computational Chemistry at New York University Shanghai, Shanghai, 200062, China*

* To whom correspondence should be addressed: xwwang@zjut.edu.cn (X.W.); xiaohe@phy.ecnu.edu.cn (X.H.)

Content

1. Illustration of the EE-GMFCC fragmentation scheme
2. Calculated excitation energies as a function of the distance threshold
3. Calculated transition electric dipole moment (TEDM) at the TD-ωB97X/6-31G* level for different model systems using the truncated full-system and EE-GMFCC method
4. The relative excitation energies for different configurations of the RNA system (pdb id: 6C63) predicted by the EE-GMFCC method and truncated full-system calculations at the TD-ωB97X/6-31G* level
5. The relative excitation energies for different RNA systems predicted by the EE-GMFCC method and truncated full-system calculations at the TD-ωB97X/6-31G* level
6. Correlation of the calculated excitation energies between the EE-GMFCC (1B and 2B) method and truncated full-system calculations
7. Comparison of the calculated excitation energies for a series of two-body fragments between the full QM method and QM/MM method

1. Illustration of the EE-GMFCC fragmentation scheme

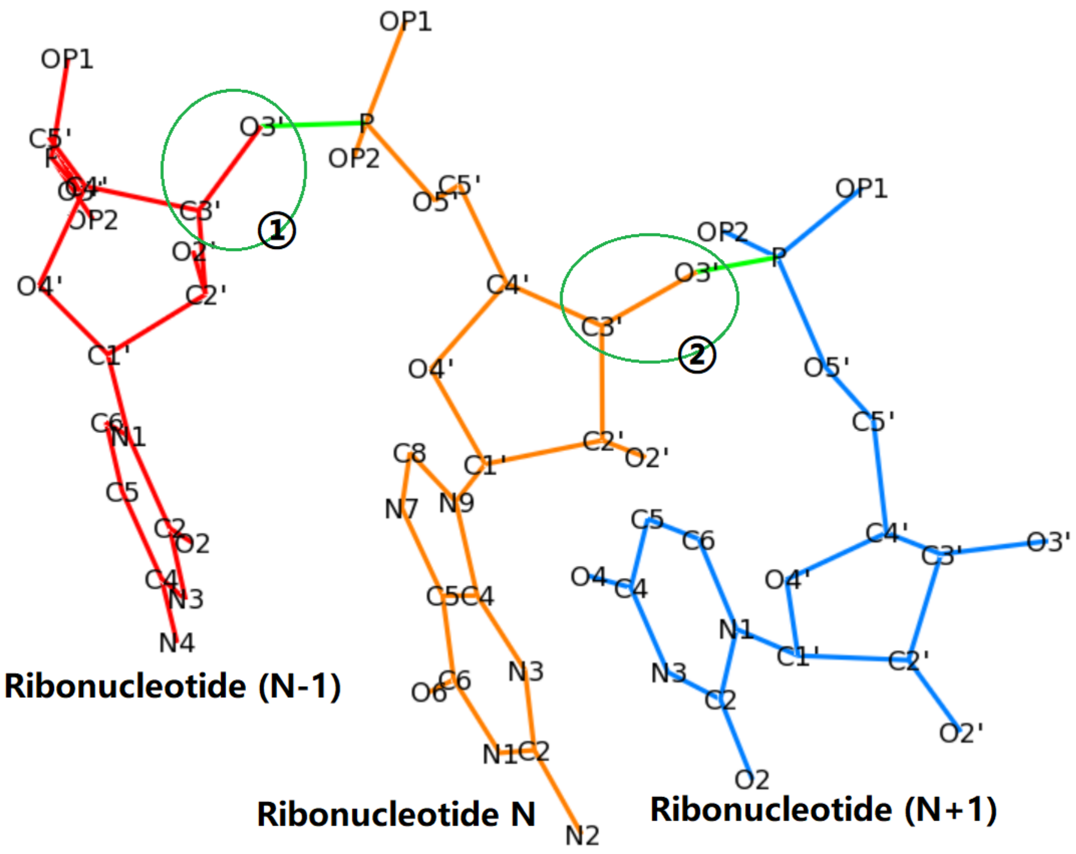

Figure S1. Illustration of the EE-GMFCC scheme for the *N*th fragment (it is shown in orange) in which the chemical bonds are cut. The cutting chemical bonds are circled in green color and marked as ① and ②, including the bond between C3 and O3 atom in the (*N-1*)th ribonucleotide (it is shown in red) and the bond between C3 and O3 atom in the *N*th ribonucleotide. The H atom was utilized to saturate the dangling bond caused by the treatment of the fragmentation scheme, and the bond lengths were set to 0.96 Å for the O-H bond and 1.09 Å for the C-H bond, respectively.

2. Calculated excitation energies as a function of the distance threshold

Table S1. Calculated excitation energies by the EE-GMFCC method using different λ_{2b} for two-body QM calculations of the model system of fullsys(7) constructed with λ_{FS} = 7 Å. The result based on the truncated full-system QM/MM calculation was labeled as FS(λ_{FS} = 7 Å). The calculations were performed at the TD-HF/6-31G* and TD-ωB97X/6-31G* levels, respectively. λ = 0 Å denotes that only fluorophore is treated by the QM method and the rest of the model system was described by the MM method using atomic charges.

Method	λ_{2B}	EE-GMFCC	Deviation[a]	FS(λ_{FS} = 7 Å)
TD-HF/6-31G*	0 Å	3.0896	0.088	3.0019
	3 Å	2.9938	-0.008	
	4 Å	2.9938	-0.008	
	5 Å	2.9917	-0.010	
	6 Å	2.9954	-0.006	
	7 Å	2.9943	-0.008	
TD-ωB97X/6-31G*	0 Å	2.6651	0.103	2.5610
	3 Å	2.5631	0.001	
	4 Å	2.5631	0.001	
	5 Å	2.5615	-0.001	
	6 Å	2.5668	0.004	
	7 Å	2.5668	0.004	

[a]Deviation of excitation energy between EE-GMFCC(2B=λ) and FS(λ_{FS} = 7 Å) for TD-HF/6-31G* and TD-ωB97X/6-31G* calculations, respectively.

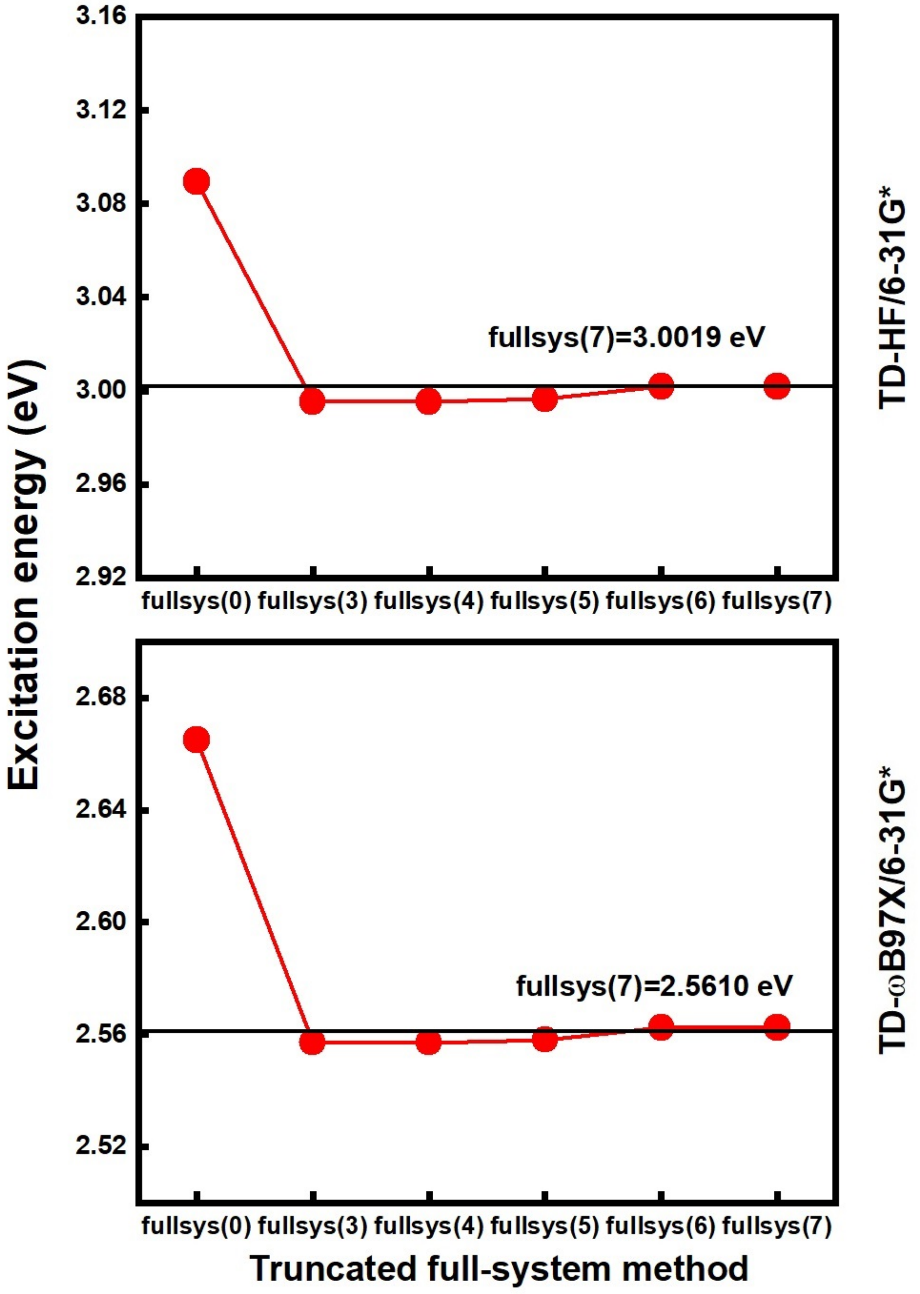

Figure S2. Predicted relative excitation energies (red line) of the truncated full-system calculations as a function of the distance threshold of λ_{FS} at the TD-HF/6-31G* and TD-ωB97X/6-31G* levels, respectively. The calculated excitation energy of the fullsys(7) system constructed using λ_{FS}= 7 Å is taken as the reference (black line).

3. Calculated transition electric dipole moment (TEDM) at the TD-ωB97X/6-31G* level for different model systems using the truncated full-system and EE-GMFCC method

Table S2. The calculated transition electric dipole moment (TEDM) using EE-GMFCC and truncated full-system calculations at the TD-ωB97X/6-31G* level in x (μ_x), y (μ_y), and z (μ_z) directions, respectively. ω was the calculated excitation energy with the largest oscillator strength *f*.

$\lambda_{2B/FS}$(Å)[a]		μ_x (a.u.)	μ_y	μ_z	ω(eV)	*f*
	1B	3.2504	0.9804	0.8142	2.6651	0.7959
3	2B	2.9886	1.0098	0.7286	2.5631	0.6583
	fullsys(3)	2.8929	0.9450	0.7398	2.5642	0.6162
5	2B	3.0066	0.9485	0.6932	2.5615	0.6539
	fullsys(5)	2.9047	0.9023	0.6946	2.5612	0.6108
6	2B	2.8318	0.9589	0.6546	2.5668	0.5891
	fullsys(6)	2.7312	0.8902	0.6794	2.5605	0.5466
7	2B	2.7829	0.9591	0.6710	2.5668	0.5732
	fullsys(7)	2.7086	0.8833	0.6912	2.5610	0.5392

[a]The same value of the λ_{FS} was employed for the λ_{2B} in the EE-GMFCC calculation.

4. The relative excitation energies for different configurations of the RNA system (pdb id: 6C63) predicted by the EE-GMFCC method and truncated full-system calculations at the TD-ωB97X/6-31G* level

Table S3. Predicated excitation energies for 10 different configurations of the RNA system (pdb id: 6C63, chain A) generated from 100 ns MD simulation using the EE-GMFCC method and truncated full-system calculation. The calculations were performed at the TD-ωB97X/6-31G* level. λ_{FS} of 4 Å was employed for constructing the truncated full system and λ_{2B} of 4 Å was utilized to tackle the two-body QM calculation in the EE-GMFCC method.

Snapshots	1B (eV)	2B (eV) (λ=4 Å)	Fullsys(4)	Deviation 1[a] (eV)	Deviation 2[b] (eV)
1	2.863	2.690	2.746	0.117	-0.056
2	2.793	2.628	2.668	0.125	-0.040
3	2.891	2.832	2.773	0.118	0.059
4	2.871	2.777	2.780	0.091	-0.003
5	2.609	2.595	2.442	0.167	0.153
6	2.840	2.730	2.755	0.085	-0.025
7	2.917	2.674	2.785	0.132	-0.111
8	2.844	2.770	2.791	0.053	-0.021
9	2.709	2.578	2.659	0.050	-0.081
10	2.727	2.634	2.650	0.077	-0.016
MUD[c]				0.101	0.056

[a]Deviation of the excitation energy between the EE-GMFCC(1B) and truncated full-system calculations (λ=4Å).

[b]Deviation of the excitation energy between EE-GMFCC(2B) and truncated full-system calculations (λ=4Å).

[c]MUD denotes the mean unsigned deviation.

5. **The relative excitation energies for different RNA systems predicted by the EE-GMFCC method and truncated full-system calculations at the TD-ωB97X/6-31G* level**

Table S4. Predicated excitation energies for 10 different RNA systems using the EE-GMFCC method and truncated full-system calculations. Similar to that of Table S4, the calculations were performed at the TD-ωB97X/6-31G* level, and λ_{FS} of 4 Å and λ_{2B} of 4 Å were employed.

PDB id	1B (eV)	2B (eV) (λ=4Å)	Fullsys(4)	Difference1[a] (eV)	Difference2[b] (eV)
6UP0, chain-C	2.983	2.784	2.729	0.254	0.055
6UP0, chain-D	3.039	2.728	2.847	0.192	-0.119
6E84	3.132	3.238	3.150	-0.018	0.088
5BJO	3.120	2.880	2.932	0.188	-0.052
6E8S	3.230	2.929	2.912	0.318	0.017
6V9D, chain-E	3.084	2.978	2.883	0.201	0.095
MUD				0.195	0.071

[a]Similar to that of Table S3
[b]Similar to that of Table S3

6. Correlation of the calculated excitation energies between the EE-GMFCC (1B and 2B) method and truncated full-system calculations

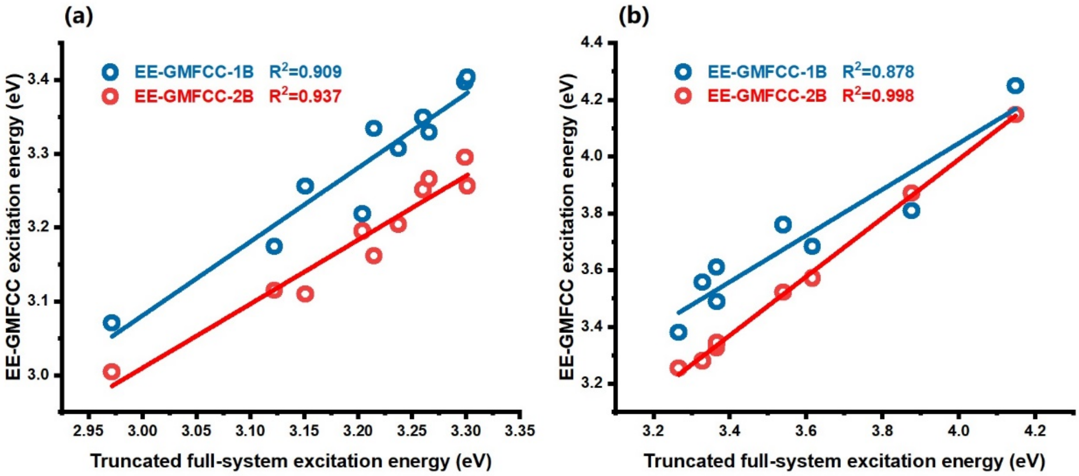

Figure S3. Correlation of the calculated excitation energies between EE-GMFCC (1B and 2B) method and truncated full-system calculations at the TD-HF/6-31G* level for (a) 10 different configurations of 6C63 selected from 100 ns MD simulation, and (b) 8 different fluorescent RNA-aptamer systems.

7. Comparison of the calculated excitation energies for a series of two-body fragments between the full QM method and QM/MM method

Table S5. Comparison of the calculated excitation energies for a series of two-body fragments between the full QM method and QM/MM (ESP and ff99OL3 charges) method[a]. The fragment structures were constructed using the GMFCC fragmentation scheme for the systems of 6UP0 chain-C and 6C63. The QM calculations were performed at the TD-HF/6-31G* level. "Fluorophore only" denotes the calculated excitation energy for the isolated fluorophore molecule. In the QM/MM calculations, the fluorophore molecule was partitioned into the QM subsystem, and the adjacent ribonucleotide was partitioned into the MM subsystem and represented by the point charges. The ESP charges used in the QM/ESP treatment were taken from the corresponding full QM calculation. The charges taken from the ff99OL3 force field were used in the QM/OL3 calculations.

TD-HF/6-31G*	Full QM (eV)	QM/ESP (eV)	QM/OL3 (eV)
6UP0 chain-C			
Fluorophore only	3.416		
Fluorophore-G10	3.394 (-0.022)	3.444 (0.028)	3.459 (0.043)
Fluorophore-G14	3.101 (-0.315)	3.195 (-0.221)	3.163 (-0.253)
Fluorophore-A15	3.216 (-0.200)	3.309 (-0.107)	3.267 (-0.149)
Fluorophore-G19	3.532 (0.116)	3.517 (0.101)	3.497 (0.081)
Fluorophore-U22	3.523 (0.107)	3.517 (0.101)	3.545 (0.129)
6C63			
Fluorophore only	3.307		
Fluorophore-A12	3.274 (-0.033)	3.308 (0.001)	3.270 (-0.037)
Fluorophore-G13	3.556 (0.249)	3.548 (0.241)	3.558 (0.251)
Fluorophore-A17	3.363 (0.056)	3.372 (0.065)	3.368 (0.061)
Fluorophore-G18	3.324 (0.017)	3.328 (0.021)	3.337 (0.030)
Fluorophore-A22	3.330 (0.023)	3.329 (0.022)	3.326 (0.019)
Fluorophore-A23	3.295 (-0.012)	3.295 (-0.012)	3.301 (-0.006)
Fluorophore-G29	3.137 (-0.170)	3.145 (-0.162)	3.161 (-0.146)

[a]The numbers in brackets are deviations of the excitation energies between various calculations (full QM or QM/MM) and "fluorophore only".
